# Tinea Capitis Kerion Type in Three Siblings Caused by Nannizzia Gypsea

**DOI:** 10.7759/cureus.55485

**Published:** 2024-03-04

**Authors:** Jesús Iván Martínez-Ortega, Arely Gissell Ramirez Cibrian, Ilse Fernández-Reyna, Carlos Enrique Atoche Dieguez

**Affiliations:** 1 Dermatology, Instituto Dermatológico de Jalisco, Zapopan, MEX; 2 Medical Benefits, Universidad Autónoma de Campeche, Campeche, MEX; 3 Mycology, Centro Dermatológico de Yucatán, Mérida, MEX

**Keywords:** terbinafine, pruritic lesions, ectoendotrix parasitization, nannizia gypsea, pseudo-alopecic plaques

## Abstract

This case report describes a rare occurrence of tinea capitis kerion type caused by *Nannizzia gypsea* in three siblings. The clinical presentation included pseudo-alopecic plaques with a dirty appearance, erythema, and honey-like crusts. A direct examination revealed ecto-endothrix parasitization in the hair shaft. Shared use of a comb among the siblings was suspected as the mode of transmission. Treatment with oral terbinafine resulted in a complete resolution. Systematic epidemiological surveys on *N. gypsea* tinea infections are scarce, and preliminary data from our center indicated a higher prevalence. The literature review identified five reported cases of *N. gypsea*-induced tinea capitis.

## Introduction

Tinea capitis, a fungal infection affecting the scalp, is primarily observed in the pediatric population and is instigated by two main fungal species:* Trichophyton* and *Microsporum* [1]. More rarely, it can be caused by *Nannizzia gypsea *(formerly *Microsporum gypseum*), a fungus that causes dermatophytosis; traditionally, it has been categorized as a geophilic dermatophyte [2]. The complex of *M. gypseum *consists of three anamorphic species: *M. gypseum*, *Microsporum fulvum*, and *Microsporum incurvatum*. From a clinical perspective, *M. gypseum* and* M. fulvum* are particularly important as they are known to cause tinea infections in humans [3]. They are opportunistic pathogens that hold epidemiological importance in causing tinea capitis [3]. The development of tinea capitis is influenced by factors specific to the host and the environment, resulting in clinical manifestations such as subtle hair loss accompanied by scalp scaling, alopecia with scaly patches [1], or, in certain instances, a kerion, which is a subtype of tinea capitis characterized by a painful, inflamed, crusty mass and is often associated with purulent drainage and regional lymphadenopathy [2].

## Case presentation

**Figure 1:**
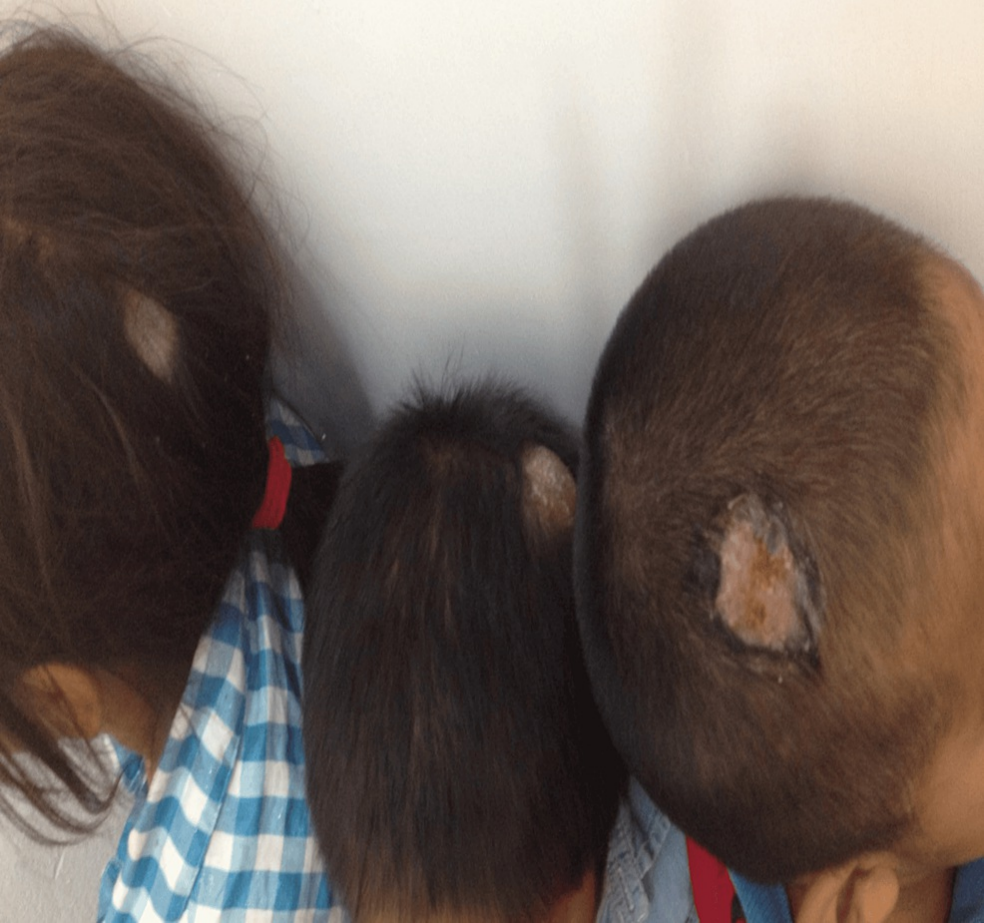
Siblings with tinea capitis This figure depicts the clinical manifestation of tinea capitis in three siblings, showcasing pseudo-alopecic plaques in the occipital and parietal regions. The plaques exhibit characteristics such as a dirty appearance, erythema, edema, and honey-like crusts.

Two male siblings aged four and five years, along with their six-year-old sister, were brought to the clinic by their father due to similar pruritic lesions on their heads persisting for a month (Figure 1).

**Figure 2:**
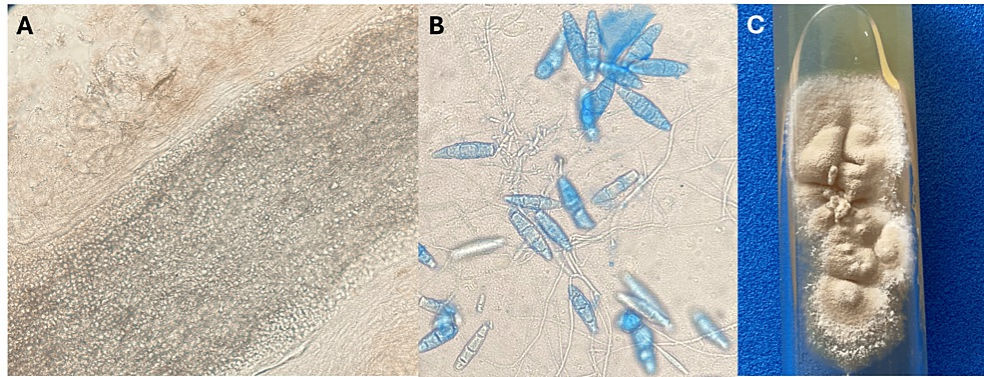
Mycologic examination Panel A illustrates the ecto-endothrix parasitization observed in the scraping samples. Panel B details the microscopic morphology of *N. gypsea* cultures stained with lactophenol blue. Sparse macroconidia with thin walls, less than six locules, blunt ends, hyaline hyphae, and limited microconidia are observed. These microscopic details contribute to the identification of the pathogenic agent. Panel C presents the macroscopic appearance of *N. gypsea *cultures on Sabouraud agar at 12 days. The colonies display a beige color, granular texture, and an appearance reminiscent of “wet soil,” providing relevant information about the fungus’s growth characteristics.

Upon examination, pseudo-alopecic plaques, measuring approximately 3 cm in diameter, were observed in the occipital and parietal regions. These plaques presented with a dirty appearance and exhibited erythema, edema, and honey-like crusts. Additionally, all three children showed palpable bilateral cervical lymphadenopathy. A direct examination revealed ecto-endothrix parasitization in the hair shaft in all three cases. Upon further inquiry with the mother, it was disclosed that the family did not have pets, but the siblings shared a comb. Consequently, oral terbinafine 125 mg was initiated daily for one month. The mycological examination suggested *N. gypsea*, and a complete clinical resolution was observed during the follow-up (Figure 2).

## Discussion

Systematic epidemiological surveys on *N. gypsea* tinea infections are exceedingly scarce in the global literature. The reported prevalence varies, but generally hovers around 1%. Even rarer are cases of tinea capitis caused by* N. gypsea* [3]. Preliminary data from our mycological center in Yucatan, Southern Mexico, across 22 years revealed a higher prevalence of 3%, accounting for 12 cases out of 479. We conducted a literature search for clinical cases of tinea capitis caused by *N. gypsea*, and the details of the five identified cases are presented in Table 1.

**Table 1:** Summary of clinical cases of tinea capitis caused by Nannizzia gypsea This table outlines reported clinical cases of tinea capitis attributed to *N. gypsea*, detailing the country of occurrence, the associated source of exposure (e.g., animal or fomite), the diagnostic method employed, and the corresponding treatment modalities. NA, not available

Age	Country	Source	Diagnosis	Treatment	References
Two years	Italy	Dog	Molecular identification	Oral griseofulvin and topical isoconazole	[4]
Three years	Italy	NA	Mycological identification	Oral griseofulvin	[5]
Six years	Spain	Probable horse	Mycological identification	Oral terbinafine and topical ketoconazole	[6]
Two years	Japan	Belt	Molecular identification	Topical luliconazole	[7]
Ten years	Japan	NA	Molecular identification	Oral itraconazole	[2]

Although newer agents have demonstrated greater efficacy in treating tinea capitis overall, griseofulvin remains particularly effective, especially in cases caused by *Microsporum*. It has served as the standard treatment since 1958 and is derived from *Penicillium griseofulvum*, disrupting the formation of mitotic spindle microtubules [8]. Unfortunately, griseofulvin is not available in our medical setting; thus, we provided terbinafine with a complete resolution.

Reports suggest transmission of zoophilic dermatophytes causing tinea capitis through fomites, such as *Microsporum canis*, through an electric razor [9] and* N. gypsea* through a belt [7]. In our case, we suspect that a shared comb is the likely causal agent, given its shared use among the three siblings. Notably, there are limited reports of tinea capitis affecting siblings [10], and specifically, there is scarce literature on *N. gypsea*, causing tinea capitis in siblings. Investigating the common factors in such occurrences is epidemiologically intriguing.

Even though the *N. gypsea* presents specific morphological characteristics identifiable by conventional mycological examination, we cannot be certain of the species unless molecular-based techniques are performed, and this represents a limitation in the case presented.

## Conclusions

Tinea capitis, caused by *N. gypsea*, is a rare but noteworthy condition. Our case highlights the importance of considering shared items, such as combs, in the transmission of dermatophytes among siblings. While newer antifungal agents show efficacy, griseofulvin remains effective, and the lack of its availability in our setting led to the use of terbinafine. Further epidemiological studies are warranted to understand the prevalence and transmission dynamics of *N. gypsea*-induced tinea capitis.
